# Preventing rheumatoid arthritis: Preferences for and predicted uptake of preventive treatments among high risk individuals

**DOI:** 10.1371/journal.pone.0216075

**Published:** 2019-04-25

**Authors:** Mark Harrison, Luke Spooner, Nick Bansback, Katherine Milbers, Cheryl Koehn, Kam Shojania, Axel Finckh, Marie Hudson

**Affiliations:** 1 Faculty of Pharmaceutical Sciences, University of British Columbia, Vancouver, Canada; 2 Centre for Health Evaluation and Outcome Sciences, St. Paul’s Hospital, Vancouver, Canada; 3 Arthritis Research Canada, Richmond, Canada; 4 School of Population and Public Health, University of British Columbia, Vancouver, Canada; 5 Arthritis Consumer Experts/JointHealth, Vancouver, Canada; 6 Division of Rheumatology, University of British Columbia, Vancouver, Canada; 7 Division of Rheumatology, University of Geneva, Geneva, Switzerland; 8 Division of Rheumatology, Jewish General Hospital and Lady Davis Institute, and Department of Medicine, McGill University, Montreal, Canada; London School of Hygiene and Tropical Medicine, UNITED KINGDOM

## Abstract

**Objective:**

To understand preferences for and estimate the likely uptake of preventive treatments currently being evaluated in randomized controlled trials with individuals at increased risk of developing rheumatoid arthritis (RA).

**Methods:**

Focus groups were used to identify key attributes of potential preventive treatment for RA (reduction in risk of RA, how treatment is taken, chance of side effects, certainty in estimates, health care providers opinion). A web-based discrete choice experiment (DCE) was administered to people at-risk of developing RA, asking them to first choose their preferred of two hypothetical preventive RA treatments, and then between their preferred treatment and ‘no treatment for now.’ DCE data was analyzed using conditional logit regression to estimate the significance and relative importance of attributes in influencing preferences.

**Results:**

Two-hundred and eighty-eight first-degree relatives (60% female; 66% aged 18–39 years) completed all tasks in the survey. Fourteen out of fifteen attribute levels significantly influenced preferences for treatments. How treatment is taken (oral vs. infusion β0.983, p<0.001), increasing reduction in risk of RA (β0.922, p<0.001), health care professional preference (β0.900, p<0.001), and avoiding irreversible (β0.839, p<0.001) or reversible serious side effects (β0.799, p<0.001) were most influential. Predicted uptake was high for non-biologic drugs (e.g. 84% hydroxycholoroquine), but very low for atorvastatin (8%) and biologics (<6%).

**Conclusion:**

Decisions to take preventative treatments are complex, and uptake depends on how treatments can compromise on convenience, potential risks and benefits, and recommendations/preferences of health care professionals. This evidence contributes to understanding whether different preventative treatment strategies are likely to be acceptable to target populations.

## Introduction

Rheumatoid arthritis (RA) is thought to develop through “multiple hits”,[1] with genetic and environmental risk factors,[2] followed by antibodies such as rheumatoid factor (RF) and anti-citrullinated protein antibodies (ACPA),[3–5] that accumulate during an “at-risk” pre-clinical phase. This pre-clinical phase lasts 3–5 years before culminating in clinical disease and can be described in five phases.[6–9] Asymptomatic phases (A-C) depend on whether individuals have genetic (Phase A) or environmental (Phase B) risk factors, or systemic autoimmunity associated with RA (Phase C). In Phase D, people first develop symptoms such as joint pain but do not have clinical arthritis. In Phase E individuals are described as having unclassified arthritis and in Phase F, RA. Increasingly, it is thought these pre-clinical phases offer a window of opportunity for potential preventive treatment.[10]

Several randomized controlled trials (RCTs) are currently exploring early intervention with traditional and biologic disease-modifying anti-rheumatic drugs (DMARDs) to prevent RA in people at increased risk for developing the disease (i.e., Phase C[11,12]) and transition from arthralgias to RA (i.e. Phase D[13–16]). This ongoing research in pre-clinical RA assumes that asymptomatic people, predicted to be at high-risk of RA (i.e. Phase C), would be willing to accept the risks and inconvenience of a treatment trying to prevent a disease that they may not subsequently develop. To date, only one small study has elicited preferences of people at-risk of developing RA for hypothetical preventive treatments, finding that preventive treatment would have to offer a high reduction in the risk of developing RA with low-risk of serious side effects.[17] Though informative, the small sample size limits the number of features that can be included, limiting the ability to predict uptake of individual treatments or explore subgroups with different preferences. Further studies with larger sample sizes, in different countries, are still needed to explore preferences and heterogeneity of prefrences of at-risk individuals for preventive treatment, and how these may affect uptake.

As uptake of treatment is critical for the success of preventive treatment, the aim of this study was to (1) understand preferences and trade-offs of asymptomatic, at-risk populations for features of preventive treatments, and (2) predict uptake of preventive treatments currently under study.

## Methods

A web-based discrete choice experiment (DCE) survey was administered to a sample of self-reported first-degree relatives (FDRs) of RA patients from the USA. FDRs represent individuals at an elevated risk of RA.[18,19] DCEs, developed in market research, assume any product, including health care interventions, can be described by characteristics (attributes) and that value of products depends on the levels of these characteristics.[20] An RA-related treatment attribute might be how treatment is taken, and levels could be oral, injection or infusion.[21] DCEs are useful where products or services do not exist (i.e. before launch). DCEs are underpinned by random utility theory. When choosing between options, individuals are assumed to assign a perceived utility (or attractiveness) to each alternative which depends on characteristics of both the alternative and the individual, and choose the one with the highest perceived utility. Random utility theory acknowledges that the utility people assign to an alternative cannot be measured (and must be treated as a random variable) meaning it is not possible to predict with certainty which alternative people will choose. Instead, random utility theory attempts to predict the probability that alternative A will be preferred to alternative B, and that this choice is proportional to the degree to which alternative A is valued more (has a higher utility) than alternative B.[22]

FDRs were recruited through Amazon’s Mechanical Turk (MTurk) platform. FDR status was determined in two-stages. Respondents self-reported whether they or an FDR (i.e. parent, sibling, adult child) had one or more of a list of chronic conditions lincluding RA. The list included clinical lycanthropy, an extremely rare condition, to screen out individuals who reported all conditions. Those who did not report RA but had an FDR with RA were invited to take the full survey. The survey included a series of questions about whether their FDR had physician-confirmed RA, and was/is taking a drug to treat RA (e.g. methotrexate or a biologic drug); those indicating yes to both questions were invited to take the survey. Each respondent received approximately $2 for completing the survey. As with a previous DCE of similar length, we excluded those who completed the entire survey in less than three minutes.[21] Ethical approval was granted by the University of British Columbia behavioural ethics board (H15-01948).

### Survey development

The DCE survey was created using five key attributes of preventive treatments identified in focus groups [23] as recommended [24,25] (Table 1). Five focus groups were conducted, two with FDRs, two with patients, and one with rheumatologists. Focus group discussions were formally analyzed for themes using framework analysis. The full analysis and derivation of attributes is described elsewhere.[23] Levels for each attribute were based on published literature, patient-information for current RA treatments being studied as prevention,[21,26] and the qualitative analysis (Table 1). An opt-out question was included to reflect that choosing not to take treatment is a realistic alternative. The survey was piloted internally for language, formatting and coding errors, and in a general population pilot sample (n = 200). Analysis of responses from the pilot sample using conditional logit analysis (see Analysis, below) resulted in coefficients of preferences for attribute levels that were logically ordered (i.e. a very rare, reversible, serious side effect was preferred to an uncommon, irreversible serious side effect) and consistent with a priori expectations based on previous RA treatment decision-making DCEs.[21,26–28]

**Table 1:** Summary of derivation of attributes and levels within the DCE.

Attribute	Presentation of attribute in survey		Development of attribute and levels	
	Attribute description	Attribute level	Representative quote from qualitative study to derive attributes	Rationale for Level
The absolute reduction of the risk of developing rheumatoid arthritis, comparing the predicted risk without and with treatment	1. Reduction in risk of RA from 60 in 100 to…	1. 44 in 100 2. 34 in 100 3. 24 in 100	“[I would consider testing] if there were perhaps a treatment that were extremely preventative and very effective at lessening the risk of developing such a disease.”–First-degree relative	Risk of RA and Risk Reduction with Treatment [4,12–14,16,29–32]
Whether treatment is given as an infusion, injection, tablet. The frequency of administration.	2. The way you take the treatment	1. IV/slow drip, twice, 15 days apart, repeated once (2 doses total) 2. Injection, once weekly for one year 3. Oral pill, once per day for one year	“You know, I went to Europe last year with my wife. We were gone for, you know, half a year. Now if I wasn’t able to do that because I had to go to a specific doctor twice a week to get this thing, no thanks. I’m good.”—First-degree relative	Based on the dosing and administration of potential preventive treatment options from rheuminfo.com [33] and consultation with clinical experts (KS, MHu)
The risk of a side effect from treatment	3. Chance of side effects	1. Common minor side effect, reversible; Uncommon serious side effect, not reversible 2. Common minor side effect, reversible; Very rare serious side effect, reversible 3. Common minor side effect, reversible	“Especially because of watching my mom with prednisone, if there’s anything that increases the mental risk that would be like huge for me.”—First-degree relative	Based on the side effect profiles of preventive treatments from rheuminfo.com[33] and consultation with clinical experts (KS, MHu)
How certain is the evidence about the risks and benefits of the treatment as a preventive option	4. Certainty in estimates	1. Very little: The true effect is likely to be substantially different from the estimate of effect. 2. Limited: The true effect may be substantially different from the estimate of the effect. 3. Moderate: the true effect is likely to be close to the estimate of the effect, but there is a chance that it is substantially different.	“Whether there was enough evidence to show that that treatment actually has a chance of preventing.”–Patient	Based on descriptions to communicate the quality of evidence published by the GRADE Working Group[34]
Whether health care professional is likely to support the use of the treatememt as a preventive option	5. Health care professional opinion	1. Your health care professional would not prefer this treatment. 2. Your health care professional is indifferent about this treatment. 3. Your health care professional would prefer this treatment.	“I think that I also have a lot of trust at this point in what health care professionals say. And a lot of my own opinions, and ultimately in the end, like it would be my own opinion, but I just think a lot of my own opinion would come from what the doctor said”—First-degree relative	Attribute elicited through qualitative framework analysis and described in a way that briefly indicates whether the physician prefers this option, does not, or is indifferent

**Fig 1:**
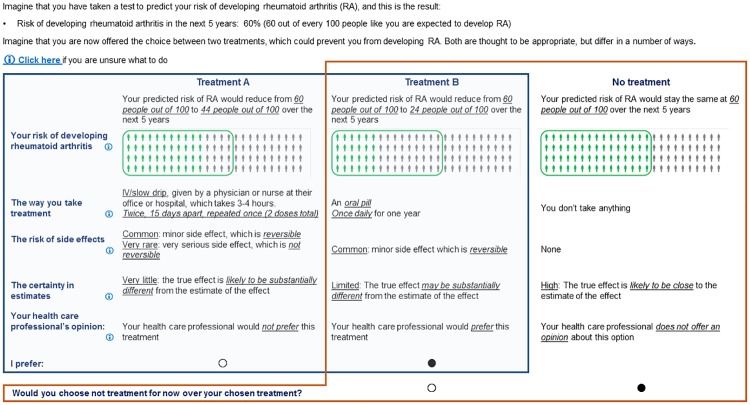
Example choice set completed by respondents. Footnote: Blue box–Part one, forced choice question, the participant chooses their preferred treatment between treatment A and treatment B; Orange box—Part two, opt out component, the participant decides whether they would take their preferred treatment of no treatment; ‘Click here’ allows the participant to view the entire informational video which was provided as part of the background instructions one more time; ‘i’ allows the participant to see the segemt of the information video relating to that particular attribute one more time; Underlined text within treatment A, treatment B, or no treatment descriptions is for emphasis only.

A DCE with 5 attributes, each with 3 levels, describes 243 combinations of levels, too many for participants to complete. Experimental designs identify a valid, reduced sample of choices for participants to complete which, if designed correctly, still answer the intended questions. A statistical program (SAS 9.1.[35]) was used to create a D-efficient design with 18 choices, these choices were blocked into 2 different versions of the survey, each with 9 questions (for more detail: S1 File). The questions (proposed treatments) selected for each version of the survey were selected based on balancing orthogonality, level, and overlap. Each participant was randomly assigned one version of the survey. In answering the survey, each participant sees 9 different choice sets (an example choice set: Fig 1). Each choice set asks them to choose their preferred treatment between two options, which were labelled Treatment A and Treatment B, and then between their preferred treatment and ‘no treatment for now.’

Before making choices, participants received background information on symptoms and complications of RA and possible preventive treatments and written and video instructions showing how to interpret information and complete the survey (survey background and instructions: S1 File). Participants also answered questions about demographic and socioeconomic characteristics.

The risk of developing RA was repeated before each choice question in the survey. The risk was set at 60 in 100 (60%) over the next five years to reflect available estimates. Each question had the same dual-response opt-out format. First participants chose their preferred preventive treatment out of treatment A or B. Next, they chose between their preferred treatment and ’no treatment for now.' The attribute levels which represented the ’no treatment’ (e.g. risk of developing RA 60 in 100 (60%) over the next five years, see red box: Fig 1) were also shown. We used a dual-response format as evidence suggests they provide better estimates of the proportion of times people would do nothing (‘opt-out’) in real situations.[36,37] Dual-response formats also may be a more efficient than standard opt-outs and improve model estimation, and brtter allow to represent diverse preferences to be represented where significant preference heterogeneity is expected.[36,37]

### Analysis

Briefly, the analysis approach was as follows (for more detail: S1 File). A conditional logit model was used to analyze responses about whether treatment A or B was preferred.[22] This model assumes an individual’s utility function (preferences) can be defined by the levels of each attribute of a good or service, and that individuals choose the option which maximizes their utility. Coefficients estimated by this model represent individual preference weights for attribute levels along with statistical significance. The attribute level expected a priori to be least preferred was used as the reference category so we expected positive coefficients for all attribute levels. The larger these coefficients, the more the attribute level contributes to the overall utility of an alternative.

Trade-offs individuals make between levels of different attributes were estimated using marginal rates of substitution (MRS). This involves modeling one attribute as a continuous variable (assumed to be linear) in the model. Coefficients for all other attributes are then divided by the coefficient for this continuous variable. Resulting MRS are presented as the increased benefit (reduction in risk or RA) needed to compensate for a treatment having a less preferred level of an attribute (e.g. the additional risk of an uncommon, irreversible serious side effect compared with only a common, reversible minor side effect).

Predicted treatment uptake was estimated using responses to the second part of the dual-response question which asked whether they would take their preferred treatment (‘Yes’) or they would not take the treatment at this point in time (No’). Both options were described as profiles of attributes and levels. A logit model was used to estimate the association between the binary choice between the preferred treatment (1 = ‘Yes’) and no treatment for now (0 = ‘No’) and attribute levels of the preferred treatment. The estimated utility for a specific preventive treatment (e.g. hydroxycholoroquine) was calculated using the coefficients for the levels of each attribute best describing that treatment (Table 2).[38] The probability of uptake of a particular treatment is the utility of that option divided by the sum of utility for all available options including nothing for now. ‘Phase C’ was limited to hydroxychloroquine. ‘Phase D’ included all treatments currently under study.

**Table 2:** Attributes and levels assigned to preventive treatments currently under study.

Treatment	Attribute and level				
	1. Reduction in risk of RA from 60 in 100 to…	2. The way you take the treatment	3. Chance of side effects	4. Certainty in estimates	5. Health care professional opinion
Hydroxychloroquine	44 in 100	Oral pill, once per day for one year	Common minor side effect, reversible	Limited: The true effect may be substantially different from the estimate of the effect	Your health care professional would prefer this treatment
Abatacept	24 in 100	Injection	Common minor side effect, reversible; Very rare serious side effect, reversible	Very little: The true effect is likely to be substantially different from the estimate of effect.	Your health care professional would not prefer this treatment.
Rituximab	24 in 100	IV/slow drip, twice, 15 days apart, repeated once (2 doses total)	Common minor side effect, reversible; Uncommon serious side effect, not reversible	Very little: The true effect is likely to be substantially different from the estimate of effect.	Your health care professional would not prefer this treatment.
Methotrexate (oral)	34 in 100	Oral pill, once per day for one year	Common minor side effect, reversible; Very rare serious side effect, reversible	Limited: The true effect may be substantially different from the estimate of the effect	Your health care professional would prefer this treatment.
Methotrexate (injectable)	34 in 100	Injection	Common minor side effect, reversible; Very rare serious side effect, reversible	Limited: The true effect may be substantially different from the estimate of the effect	Your health care professional would prefer this treatment.
Atorvastatin	44 in 100	Oral pill, once per day for one year	Common minor side effect, reversible	Limited: The true effect may be substantially different from the estimate of the effect	Your health care professional is indifferent about this treatment.

Levels are subjective and assigned based on expert opinion (MHu, KS, AF). Each of the 5 attributes has 3 levels, for each treatment currently being investigated in a randomized controlled trial.

Latent class analysis was used to identify subgroups (latent classes) with distinct patterns of preferences.[39] Membership of one of these latent classes is predicted by an individual’s preferences for attribute levels. The relative importance of each attribute was calculated for each latent class by dividing the range of coefficients for each attribute by the sum of all coefficient ranges within the DCE.[40,41] Labels for each class were then provided qualitatively to summarize the attributes which were most important to each group. Differences in participant characteristics (age, sex, education, income, health insurance, ancestry and willingness to pay for preventive treatment) between the individuals assigned to different latent classes were tested using a chi-squared test. The probability of uptake of particular treatments was compared between latent classes.

## Results

Of 525 initial respondents, 288 (55%) had an FDR with physician-confirmed RA currently taking a drug for RA, meeting our inclusion criteria as FDRs. No participants were excluded for completing the survey within 3 minutes (minimum time-to-complete 3.28 minutes; mean 9.11 (SD 8.29)) and none always chose Treatment A or treatment B. The ‘no treatment for now’ option was chosen 33% of the time (for 856 of the total 2592 choices made), and 10% of participants (n = 29) always chose no treatment. The sample was primarily female (60%), aged 18–39 years (66%), and most reported European (84%) ancestry (Table 3). A minority (12%) reported no medical insurance; those with private insurance mainly had employer plans (51%), 22% had Medicare/Medicaid coverage. Most (91%) indicated that they would be willing to pay out of pocket for preventive treatment for RA.

**Table 3:** Participant characteristics of the overall sample and latent classes of respondents within the overall sample.

		Overall sample		Subgroups identified by latent class analysis†						Between class differences
		First-degree relatives n = 288		Class 1 ‘safety first’ n = 90		Class 2 ‘need reassurance’ n = 111		Class 3 ‘convenience’ n = 87		
		n	%	n	%	n	%	n	%	p
Sex:	Female	173	60%	52	58%	63	57%	58	67%	0.443
Age:	18–39	190	66%	58	80%	80	72%	52	60%	0.185
	40–59	89	31%	31	34%	28	25%	30	34%	
	60+	9	3%	1	1%	3	3%	5	6%	
Education:	Up to high school	47	16%	16	18%	12	11%	19	22%	0.173
	Some post-secondary	124	43%	36	40%	56	50%	32	37%	
	Undergrad/post-grad	117	41%	38	42%	43	39%	36	41%	
Income:	<$15,000	15	5%	7	8%	2	2%	7	7%	0.558
	$15,000 to $30,000	40	14%	11	12%	19	17%	10	11%	
	$30,000 to $50,000	79	27%	25	28%	32	29%	22	25%	
	$50,000 to $80,000	79	27%	27	30%	30	27%	22	25%	
	$80,000 to $150,000	59	20%	17	19%	22	20%	20	23%	
	≥$150,000	11	4%	1	1%	4	4%	6	7%	
	Prefer not to say	5	2%	2	2%	2	2%	1	1%	
Health Insurance:	Medicare/Medicaid	64	22%	15	17%	28	25%	21	24%	0.306
	Private (individual)	42	15%	9	10%	19	17%	14	16%	0.325
	Private (employer)	148	51%	48	53%	58	52%	42	48%	0.776
	No insurance*	35	12%	17	19%	7	6%	11	13%	0.025
Ancestry:	European	242	84%	80	89%	92	83%	70	80%	0.284
	Aboriginal	8	3%	1	1%	5	5%	2	2%	0.329
	African	14	5%	4	4%	7	6%	3	3%	0.634
	S Asian	1	<1%	-	-	1	<1%	-	-	0.449
	SE Asian	7	2%	2	2%	4	4%	1	1%	0.532
	E Asian	3	1%	1	1%	-	-	2	2%	0.286
	M Eastern	4	1%	1	1%	0	0%	3	3%	0.116
	Hispanic	24	8%	7	8%	7	6%	10	11%	0.412
Willingness to pay out of pocket for preventive treatment:	$0	27	(9%)	10	11%	5	5%	12	14%	0.042
	$200	125	(43%)	33	37%	47	42%	45	52%	
	$1000	117	(41%)	40	44%	49	44%	28	32%	
	$5000	19	(7%)	7	8%	10	9%	2	2%	

^†^ Latent-class conditional logit models were used to identify whether any unobservable subgroups could be identified within our group of FDRs based on their preference structures. Three classes (1) with preferences dominated by minimizing the risk of side effects and maximizing the amount of certainty in risk and benefit estimates (31% of the sample) labelled as ‘safety first., (2) with strong preferences for the reduction of risk of RA, mode of administration, and a treatment which preferred by the health care professional (39% of sample) labelled as ‘need reassurance.’ (3) with strong preferences for reduction in the risk of RA and less invasive modes of administration (30% of the sample) labelled ‘convenience’.

### Preferences for treatment attributes

Coefficients, representing preferences, increased in the order we expected to be favored for all attribute and were statistically significant (except ‘limited’ level of the ‘certainty in estimates’ (p = 0.233)) (Table 4). Preferences were strongest for daily oral treatment compared with two infusions (β0.983, p<0.001), followed by a reduced risk of developing RA within 5 years from 60% to 24% (β0.922, p<0.001), and treatment preferred by a health care professional compared with one that was not (β0.900, p<0.001). Preferences were also strong for either no uncommon irreversible serious (β0.839, p<0.001) or very rare reversible serious side effects (β0.799, p<0.001). Preferences for increasing certainty in benefits and risks of treatment (very little to moderate (β0.416, p<0.001)) were comparable in size to preferences for reducing the risk of developing RA within 5 years from 60% to 34% (β0.505, p<0.001).

**Table 4:** Estimated coefficients from conditional logit model.

Attribute	Attribute level	Overall sample		Marginal rates of substitution	Subgroup analysis: Latent classes		
					Class 1	Class 2	Class 3
		n = 288			n = 90 (31%)	n = 111 (39%)	n = 87 (30%)
		Coeff.	p-value		Coeff.	Coeff.	Coeff.
1. Reduction in risk of RA from 60 in 100 to…	1. 44 in 100	Ref	Ref	N/A	Ref	Ref	Ref
	2. 34 in 100	0.505	0.000	N/A	1.191	0.832	0.013
	3. 24 in 100	0.922	0.000	N/A	1.471	1.521	0.265
2. The way you take the treatment	1. IV/slow drip, twice, 15 days apart, repeated once (2 doses total)	Ref	Ref	Ref	Ref	Ref	Ref
	2. Injection	0.192	0.004	-4.1 (-6.9, -1.2)	-0.821	-0.057	1.022
	3. Oral pill, once per day for one year	0.983	0.000	-21.6 (-25.6, -17.5)	0.845	0.361	2.717
3. Chance of side effects	1. Common minor side effect, reversible; Uncommon serious side effect, not reversible	Ref	Ref	Ref	Ref	Ref	Ref
	2. Common minor side effect, reversible; Very rare serious side effect, reversible	0.799	0.000	-17.5 (-21.1, -13.8)	3.688	0.340	0.518
	3. Common minor side effect, reversible	0.839	0.000	-18.2 (-21.8, -14.6)	4.129	0.589	0.172
4. Certainty in estimates	1. Very little: The true effect is likely to be substantially different from the estimate of effect.	Ref	Ref	Ref	Ref	Ref	Ref
	2. Limited: The true effect may be substantially different from the estimate of the effect.	0.082	0.233	-1.91 (-4.9, 1.1)	-0.149	0.292	-0.073
	3. Moderate: the true effect is likely to be close to the estimate of the effect, but there is a chance that it is substantially different.	0.416	0.000	-9.2 (-12.3, -6.1)	1.728	0.596	0.003
5. Health care professional opinion	1. Your health care professional would not prefer this treatment.	Ref	Ref	Ref	Ref	Ref	Ref
	2. Your health care professional is indifferent about this treatment.	0.508	0.000	-11.2 (-14.5, -8.0)	1.358	0.663	0.666
	3. Your health care professional would prefer this treatment.	0.900	0.000	-19.8 (-23.9, -15.8)	1.481	1.346	1.022

Estimates of MRS show how much better at reducing the absolute risk of developing RA a treatments with a less preferred levels of an attribute would need to be to be equivalent to one with most favorable level. To be comparable with an oral pill, infused treatments would need to reduce the risk of RA by an additional 22% (95% CI 17%, 26%) and sub-cutaneous injections an additional 4% (95% CI 1%, 7%). Similarly, treatments with a risk of uncommon but irreversible serious side effects would need to reduce the risk of developing RA by an additional 18% (95% CI 15%, 22%), or an additional 17% (95% CI 14%, 21%) for a risk of very rare but reversible serious side effects. Treatments not preferred by health care professionals would need to offer an additional 20% reduction (95% CI 16%, 24%) in the risk of RA to be equivalent to preferred treatments, and those with very little certainty in evidence of risks and benefits would need to offer an additional 9% (95% CI 6%, 12%) reduction in the risk of RA to be equivalent to those with moderate certainty.

### Predicted uptake

**Fig 2:**
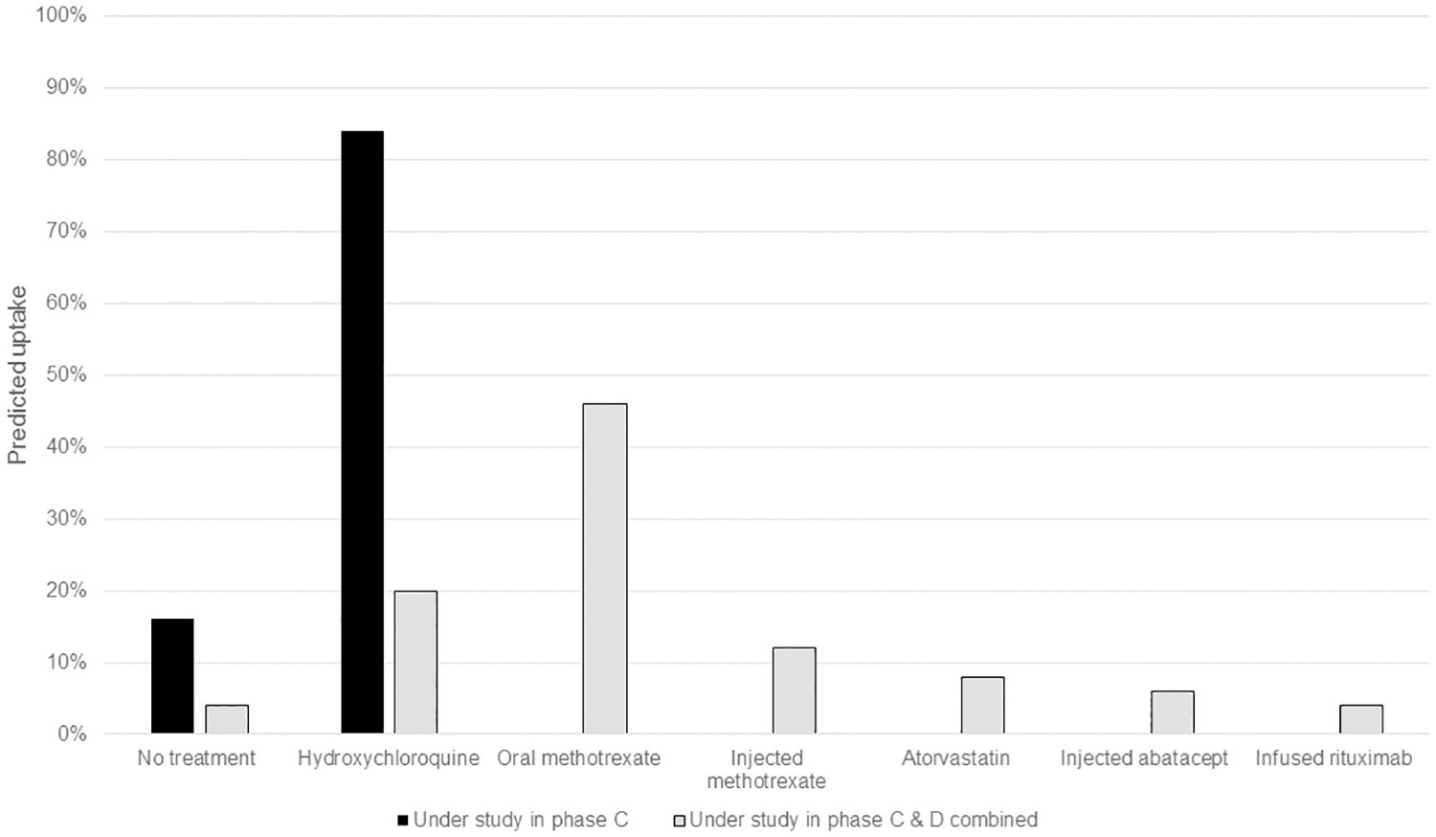
Predicted uptake: Treatments under study.

If hydroxychloroquine (Phase C), was the only option as preventive treatment (versus doing nothing), the predicted probability of uptake was 84% (Fig 2). Including abatacept, rituximab, methotrexate and atorvastatin, which are currently studied as prevention, increased the predicted probability of uptake of any treatment to 96%. In this scenario, the predicted probability of uptake was highest for oral methotrexate (46%) and hydroxychloroquine (20%), and very low for atorvastatin (8%) and biologics (abatacept (6%); rituximab (4%)).

### Subgroup preferences

**Fig 3:**
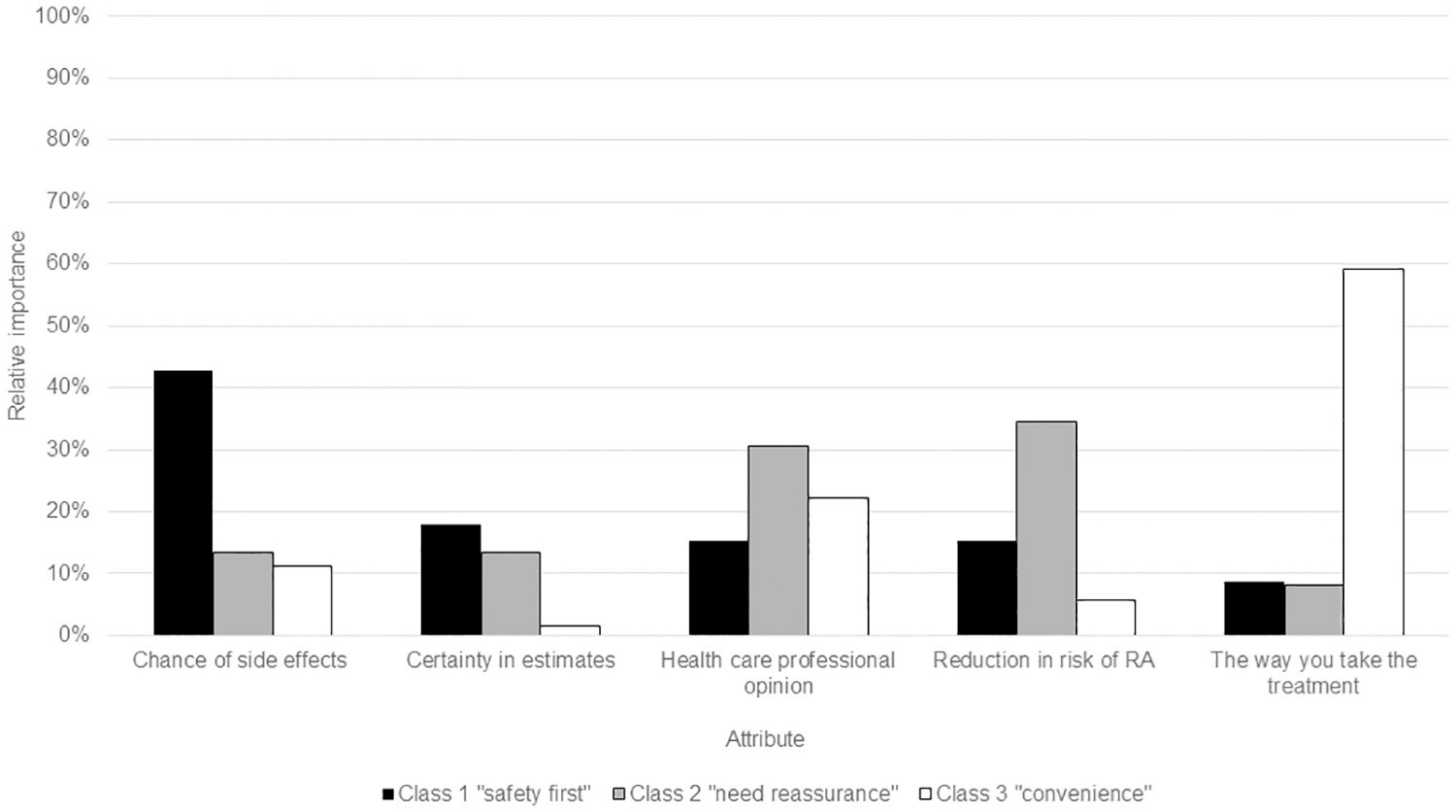
Relative importance of attributes within each of the three latent classes identified.

Three subgroups of respondents had different preferences for preventive treatment. No characteristics were associated with membership of any subgroup, aside from small but significant differences in the proportion with no health insurance (19% class one, 6% class two, 13% class three; p = 0.025) (Table 3). Preferences of class one (31% of the sample) were dominated by minimizing risks of side effects and maximizing certainty in risk and benefit estimates (Table 4 and Fig 3); this group was labelled ‘safety first.’ Group two (39% of sample) had strong preferences for reducing the risk of RA, how treatment is taken, and treatments preferred by health care professionals, and was labelled ‘need reassurance.’ Class three (30% of the sample) had strong preferences for reducing the risk of developing RA, but more importantly less invasive treatment, and was labelled ‘convenience.’

Probability of uptake varied by class; uptake of hydroxychloroquine as the only preventive treatment was higher in group two (‘need reassurance’) (96%) than groups one (‘safety first’) (64%) or three (‘convenience’) (71%).(S1 Fig) If all preventive treatments being studied were available, the proportion who would not take anything was lower in group one (‘safety first’) (10%) than group two (‘need reassurance’) (1%) or three (‘convenience’) (5%). Non-biologic DMARDs had highest predicted uptake across all groups; methotrexate ranged from 37% (group one: ‘safety first’) to 50% (group three: ‘convenience’) and hydroxychloroquine from 12% group three to 29% (group two: ‘need reassurance’). Predicted uptake of hydroxychloroquine (12%) was lower in group three (‘convenience’) than injected abatacept (15%), but predicted uptake of biologics was low for all groups (rituximab 2–4%, abatacept 7% in class one and 4% in class two).

## Discussion

Results of this study suggest asymptomatic, at-risk individuals, may be willing to take preventative treatment to reduce their risk of RA, although they have different appetites for risk, reassurance, and convenience of treatments. Importantly, our findings suggest preventive treatments preferred by at-risk individuals will not necessarily be those offering the largest reduction in risk of RA; uptake is likely to be driven as much by how treatment is taken, opinions/preferences of health care professionals, and the risk and reversibility of side effects. Assuming that treatments currently being evaluated in RCTs all offer preventive efficacy, we predict highest uptake of convenient treatments offering low-to-moderate reductions in risk of developing RA and low risks of serious side-effects. This suggests that non-biologic DMARDs like hydroxychloroquine and potentially methotrexate are more likely to be acceptable preventive options than the biologic DMARDs being tested.

We predict higher uptake of preventive treatment (86%) than the only comparable previous estimate (38%), which was based on a baseline risk of RA of 40%.[17] This previous study did predict that uptake would increase as baseline risk increased (7% at 1 in 100 risk; 38% at 40 in 100 risk). Our higher baseline risk (60 in 100 over 5 years) of developing RA[4,32] could explain our higher predicted uptake. This baseline risk was based on 5-year estimates of the risk of developing RA in individuals with a family history of RA and the presence of autoantibodies which ranged from 58% to 69%.[4,32] These estimates correspond well with the context and time frame of the “at-risk” pre-clinical phase C, where people have evidence of systemic autoimmunity associated with RA but no arthralgia, that we sought to represent. Further research is needed to understand the relationship between baseline risk and uptake of preventive treatment. Our higher uptake could also relate to our dual-response question format. However, our prediction that 86% of participants would consider hydroxychloroquine is consistent with previous conclusions that people would ‘take moderately effective treatment if the risk of a serious adverse event was very low.’[17]

Our findings have two potentially major implications. One is greater patient/stakeholder involvement in choices of treatments to study in RCTs and the magnitude of benefit required on primary endpoints. All current RCTs of preventive treatment have reduction in the number of people developing RA as their primary endpoint. Given that safety, convenience and concordance with health care professional opinions may be more important, and people are willing to trade between attributes, a greater effect size on the primary endpoint may be needed to compensate for less convenient and/or safe treatments. Conversely, relatively safe and/or convenient treatments might only need to show relatively small reductions in risk of developing RA to be acceptable. Having information on treatment preferences prior to study design stage could help to ensure that RCTs are powered to demonstrate effect sizes important to potential recipients. More patient-oriented trials could test treatments or magnitudes of benefit fulfilling these criteria, and treatments unlikely to meet patient preferences can be identified earlier.[42] Our findings suggest the hydroxychloroquine is the preferred treatment from the consumer perspective, as well as potential for methotrexate to be explored as an earlier option (based on our assumption would reduce risk of developing RA more than hydroxychloroquine). Whilst we do not know whether these options will be less effective in reducing the risk of RA than biologic DMARDs, their relative safety and convenience may be more important for RA-free people who do not want to take excessive risks or would prefer to try safer pharmacological alongside other approaches to reduce their risk of developing RA such as changing their diet or stopping smoking. Our results provide little support for biologic-drugs as preventive options; despite assuming biologics would offer the greatest risk reduction (reducing risk of RA from 60 to 24 in 100), we predict very low uptake of abatacept and rituximab (≤6%).

The second implication highlights the critical role of health care professionals in preventive treatment decisions. The health care professional’s opinion was among the most important attributes influencing preventive treatment decisions. To help people choose options that best reflect their preferences, we need to recognize trade-offs, value and relative importance that people place on features of treatments.[43] Decision support interventions could help health care professionals to elicit preferences of at-risk people and help them choose treatments consistent with those preferences.

Our study has a number of limitations. First, attributes and levels included in the survey influence preferences estimated from responses; including different attributes and levels would likely generate different results. However, the survey was developed in line with best-practice recommendations using focus groups of patients, their FDRs and rheumatologists[23] to identify relevant attributes and ensure that information was clear and easy to understand.[25] Further, selected attribute levels reflected treatments currently being studied. Secondly, out-of-pocket costs could influence preferences for preventive treatment, but was omitted. Cost, however, ranked last when we asked patients and FDR groups to prioritize potential attributes, and is also problematic to include as there is significant variation in the costs associated with medications according to whether someone has insurance coverage or not, and what is covered by insurance.[23] Thirdly, using MTurk limits our ability to determine whether respondents genuinely were FDRs of people with RA. The general public are known to confuse symptoms and diagnosis of RA with those of osteoarthritis. Our sample was also more likely younger and internet savvy; two-thirds were aged 18–39 years. However, we used a series of strict study entry criteria and, even after a pre-qualification survey, excluded 45% of our sample that we could not confidently classify as FDRs. Future, comparable, research with verifiable FDRs of RA patients with a confirmed diagnosis would be valuable. MTurk samples also have evidence of their validity[44,45], representativeness[46] and accurate/less biased responses.[47,48] Finally, as with any DCE, we cannot be sure that participants understood the tasks, made choices consistent with underlying theory, or that their responses reflected true preferences because test and treatment scenarios are hypothetical and we do not know their risk of disease (FDR status is a proxy for risk). The disconnect between stated (hypothetical) and revealed (actual) preferences (hypothetical bias) threatens the external validity of DCEs[49], but a recent systematic review and meta-analysis of DCEs concluded that DCEs generate reasonable predictions of health-related behaviors, particularly when predicting how someone will behave (e.g. take preventive treatment).[50] The review did caution that imperfect predictions from DCEs may lead to overestimates of demand for treatments, which supports our caution that our predictions of uptake could be overestimates.

Strengths of this study are that it is the first large sample of self-reported FDRs of people with RA, (most accurately described as Phase A (genetic risk factors for RA) as their autoimmunity status is unknown) to describe trade-offs between features of preventive treatments. However, preferences of this sample may not generalize to RA-naïve populations who have not observed the effects of RA on family member(s). A further strength is our design that allows us to predict uptake not only of preventive treatments currently under study, but other treatment options that may be available in future. Uptake estimates provide important parameters in future economic evaluations of the cost-effectiveness of preventive treatments, once results of ongoing trials are published. Future research to contrast predicted and actual uptake of preventive treatment in at-risk individuals will be valuable.

## Conclusion

We provide preliminary predictions of uptake of preventive treatments for RA in currently RCTs. Our findings suggest people may take preventive treatment, but reduction in the risk of developing RA is one of multiple factors influence their preferences. Furthermore subgroups exist that prioritize safety, reassurance, and convenience. Decisions to take preventive treatment are complex. Only treatments that balance these preferences will be acceptable to at-risk populations, and as preferences are not predicted by socio-demographic characteristics they may need to be elicited on an individual basis. Preferences of potential recipients should also be considered when designing RCTs to prioritize studies of interventions likely to taken by potential recipients if they meet their primary endpoint.

## Supporting information

S1 Fig Predicted uptake of treatments under study by latent class. (TIF)

S1 File Supplementary data. (PDF)

S2 File Data file. (XLSX)
