# Supplementary figures and images for: Preventing rheumatoid arthritis: Preferences for and predicted uptake of preventive treatments among high risk individuals
Source: PLoS One. 2019 Apr 25;14(4):e0216075. doi: 10.1371/journal.pone.0216075 (PMC6483264; doi:10.1371/journal.pone.0216075)

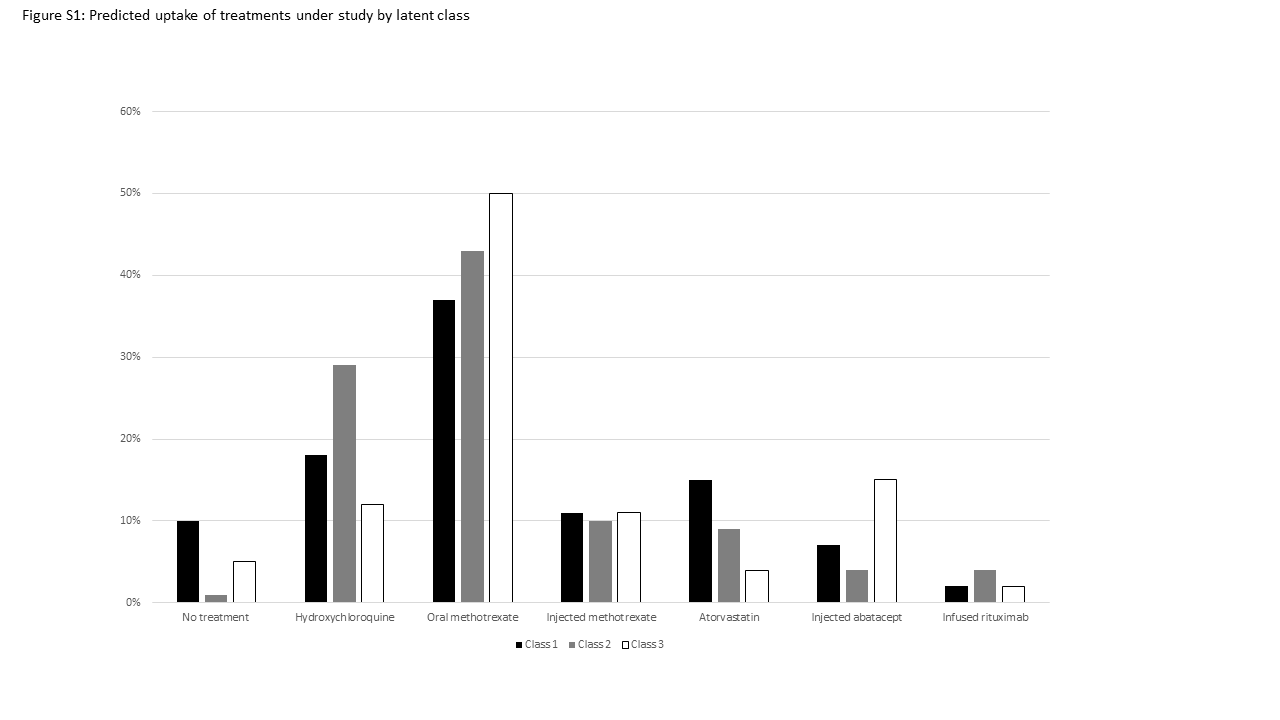

Supplement: S1 Fig — (TIF) [file pone.0216075.s001.tif]
