# Supplementary material for: Preventing rheumatoid arthritis: Preferences for and predicted uptake of preventive treatments among high risk individuals
Source: PLoS One. 2019 Apr 25;14(4):e0216075. doi: 10.1371/journal.pone.0216075 (PMC6483264; doi:10.1371/journal.pone.0216075)
Supplement: S1 File — (PDF) [file pone.0216075.s002.pdf]

S1 File: Supplementary Data

Background information and survey

Why is the survey being carried out?

The purpose of this project is to find out what people think about a range of treatment options intended to try and prevent the development of symptoms of rheumatoid arthritis. Rheumatoid arthritis is a disease in which the body's immune system mistakenly attacks healthy parts of the body, causing swelling, damage to the joints and other health problems such as problems with the heart. With early diagnosis and drug treatment, rheumatoid arthritis can be successfully managed. Rheumatoid arthritis is thought to develop by a "multiple hit" process, where many things which increase the risk of disease come into contact with each other and trigger the disease. New research is finding that it may be possible to predict who may develop rheumatoid arthritis in the long run, and that some rheumatoid arthritis treatments may help prevent the disease from developing.

We want to find out what you think about some possible ways of taking medications to try and prevent the development of rheumatoid arthritis, and how you trade off the risks and benefits. This information will be used to help us better understand what characteristics of a potential preventative treatment are important to you.

What you will be doing in the survey

On the next page, there will be a few questions to answer about yourself. After that, we will provide you with the background and objectives for the study.

We will then ask you to make some choices. You will be given a background scenario at the top, which will stay the same throughout the entire survey. The background has information on an imaginary test you have taken, which tells you your risk of developing rheumatoid arthritis (RA). It will also have information on how accurate this test is.

You will then be asked to choose between two treatment options. These options are defined by this set of features:

- Your risk of developing RA, and how much it could reduce with treatment
- The way you take the treatment
- Chance of side effects, which will include minor and major side effects
- Certainty in estimates, or how certain we are that the true effect of the medication is close to our estimate
- Your health care provider's opinion

The choices we will ask you to make

There will be two choices per scenario in the survey: first, we will ask you to choose between the two preventative treatment options (A and B) which will be given.

Then, we will ask you to choose whether you would rather stay with your preferred treatment, or choose no treatment at all for now (a small table below will remind you of what happens if you choose no treatment at all for now).

Your survey will contain 11 scenarios in total. To make viewing the survey as easy as possible, we suggest having your browser window in full screen mode.

What if I forget what to do?

Before you do the survey, there is a 5 minute informational video, which shows what the choice set will look like, and walk you through a complete scenario. We recommend that you watch this before moving on to the survey.

You can watch the video at any time by clicking "Click Here" above the choice set.

Alternatively, you can hover your mouse over the name of the feature to see a small text box describing it.

[Video]

Video script:

<display title slide>

We are going to ask you to make choices between two preventative treatments. We are only interested in your preference, so there are no right or wrong answers. This is what each choice looks like. <display slide of choice set>

The background information <highlight> tells you the result of an imaginary test which estimates how likely you are to get rheumatoid arthritis <highlight this line. This background information will be the same for all choices that you make.

Keeping in mind the background information, in Part 1 you will be asked to choose between two options, Treatment A <highlight> and Treatment B <highlight>, each with five key features <highlight the features with the arrow list>. These features will differ between the two options, and will change with each new scenario. If you hover your mouse over the label for each feature, a pop-up text appears to remind you of what it describes <demonstrate>.

The first feature <highlight box around it> tells you how your predicted risk of developing rheumatoid arthritis reduces if you took this treatment <highlight the info for one of the treatments>. Imagine 100 people, just like you. The test predicts that, without treatment, some of those people, shown in the green box <highlight>, will get rheumatoid arthritis. If they all took this treatment, and it works, some of those people would avoid getting rheumatoid arthritis <highlight>, and fewer people, now only those shaded in green <highlight those in green and the text>, would develop rheumatoid arthritis.

<highlight box around second attribute> You are then told how you would take the treatment <highlight info for one of the treatments>. All treatments last for one year.

<highlight box around third attribute> Next is the chance of side effects <highlight info for one of the treatments>, including how serious they are and if they are reversible, and

<highlight box around fourth attribute> below this, <highlight info for one of the treatments> the certainty we have in the estimates of risks and benefits is shown. Although treatments for preventing RA might include drugs that are currently used to treat the disease, we know less about their risks and benefits for prevention than we do for treating RA. As more and more people use a treatment, we find out more information that could show whether our early estimates were accurate or inaccurate.

<highlight box around fifth attribute> The last feature is <highlight info for one of the treatments> Your healthcare professional's opinion about the treatment. Whether you and a healthcare professional think the same option is best will be important for some people, but not for others.

After considering each choice, choose the treatment you prefer by clicking the button below that option <highlight here for option A or here for option B>. Remember there are no right or wrong answers, the option you choose depends on what is important to you.

Part 2 asks <highlight bottom part of survey> whether you'd prefer to take the treatment you selected or no treatment at all for now. The information here <highlight table> shows what each feature looks like if you choose no treatment.

Now that you know what it will look like, please go ahead and make these choices yourself.

If you need to re-watch this video at any point click here <highlight link>. Alternatively, you can hover your cursor over a feature to see more information about it.

Thank you.

Background

What is rheumatoid arthritis (RA)?

A disease where your immune system, which normally fights infection (such as when you get a cold), starts to attack the body, leading to widespread inflammation. Symptoms and complications of RA include:

- Your joints becoming hot, swollen, stiff, and painful
- Experiencing pain, fatigue, limited mobility and limited ability to work
- Heart disease and infections
- Shortened life expectancy

Early treatment is recommended to minimize joint damage and other complications.

Predicting who will get rheumatoid arthritis

RA is thought to develop by a "multiple hit" process. In other words, many things which increase the risk of disease come into contact with each other and trigger the disease. These include:

- Your sex, with women being several times more at risk than men
- Your family history of rheumatoid arthritis, which might suggest a genetic link
- A number of environmental triggers, for example low sun exposure (leading to low vitamin D), or pollution
- Lifestyle factors, for example smoking and poor diet

Early stages of disease can now be tested for before any symptoms are noticed or any harm occurs. This means that we may be able to predict who is at high risk of rheumatoid arthritis.

As with any predictive test, though, there is a chance that the prediction might be wrong. If that was the case, some people said to be at high-risk of rheumatoid arthritis might never get the disease and some we think to be at low risk might still get it.

DCE Task Instructions

Imagine that you have taken a test to predict your risk of developing rheumatoid arthritis (RA), and this is the result:

- Risk of developing rheumatoid arthritis in the next 5 years: 60% (60 out of every 100 people like you are expected to develop RA)

Imagine that you are now offered the choice between two treatments, which could prevent you from developing RA. Both are thought to be appropriate, but differ in a number of ways

Non-DCE survey questions

A few questions about you...

What is your age?

Select one...

Please select your gender.

- Female
- Male
- I'd prefer not to say
- Other - please specify

Do you have a physician-confirmed diagnosis of rheumatoid arthritis?

- Yes
- No
- I'm not sure

Do you have any of the following medical conditions? Please select all that apply.

- Inflammatory arthritis
- An inflammatory condition requiring immunosuppressant therapy (for example: Crohn's disease, uveitis, psoriasis)
- I do not have these
- I am not sure

Are you currently taking, or have you previously been taking, a drug to treat rheumatoid arthritis, for example, methotrexate or a biologic drug?

- Yes
- No
- I'm not sure

Have you ever had any joints replaced in your body?

- Yes
- No
- I'd prefer not to say
- I'm not sure

Do you currently, or have you ever, had to take any medications that required you to inject yourself (for example, insulin for diabetes, or a biologic drug for rheumatoid arthritis?)

- Yes
- No
- I'd prefer not to say

Do you have a family member with a diagnosis of rheumatoid arthritis that has been confirmed by a physician?

- Yes
- No
- I'm not sure

Are you aware of any family member that is currently taking, or has previously taken, a drug to treat rheumatoid arthritis, for example, methotrexate or a biologic drug?

- ○ Yes
- ○ No
- ○ I'm not sure

Which country do you live in permanently?

- ○ Canada
- ○ United States of America
- ○ Other

What is the highest level of education that you have completed?

Select one...

What is your current yearly household income?

Select one...

What healthcare coverage do you currently have? Please select all that apply.

- ☐ State or public coverage (for example, Medicare, Medicaid)
- ☐ Individual private insurance
- ☐ Private insurance through an employer
- ☐ None (I would pay out of pocket for health services)
- ☐ Other

To which ethnic or cultural groups did your ancestors belong? Please select all that apply.

- ☐ European
- ☐ South Asian (for example: Indian, Pakistani, Sri Lankan)
- ☐ Aboriginal/Native American (for example First Nations, Métis or Inuit)
- ☐ African
- ☐ Southeast Asian (for example: Vietnamese, Cambodian, Filipino)
- ☐ Middle Eastern
- ☐ East Asian (for example: Chinese, Japanese, Korean)
- ☐ Latin American/Hispanic
- ☐ I'd prefer not to say
- ☐ Other - please specify ______

Which health care professional(s) have you visited or contacted in the past year for chronic conditions such as rheumatoid arthritis? Please select all that apply.

- ☐ General physician/family doctor
- ☐ Specialist (i.e. rheumatologist)
- ☐ Licensed/registered nurse
- ☐ Pharmacist
- ☐ Physiotherapist
- ☐ Occupational therapist
- ☐ Naturopathic doctor
- ☐ None
- ☐ Other

Which health care professionals would you want to hear advice from about preventative treatments for rheumatoid arthritis? Please select all that apply.

- ☐ General physician/family doctor
- ☐ Specialist (i.e. rheumatologist)
- ☐ Licensed/registered nurse
- ☐ Pharmacist
- ☐ Physiotherapist
- ☐ Occupational therapist
- ☐ Naturopathic doctor
- ☐ Other health care professional
- ☐ None of the above

What is the maximum amount you would be willing to pay out of pocket for a year-long treatment that might prevent you from developing RA?

- ○ Nothing ($0.00)
- ○ $200.00
- ○ $1000.00
- ○ $5000.00

Who should pay for preventative strategies for diseases such as RA?

- ○ People should pay out of pocket
- ○ Private insurance providers
- ○ Public/state funded health insurance, such as Medicare/Medicaid
- ○ Government
- ○ Employers' private insurance
- ○ Other - please specify ______

Analysis of DCE responses

DCE responses were analyzed in two stages, corresponding with the two stages of the question. A conditional logit model was used to analyze data where the preferred treatment (A or B) was chosen. The conditional logit model estimates the relative strength of preferences for the levels of each attribute using the assumptions of the Random Utility Model.(2) This assumption is that, in a DCE, each individual *i* has a construct of utilities (or value) for each alternative in a choice described by a profile *k*, which can be defined as:

$U_{ik} = \mu_{ik} + \varepsilon_{ik}$

where μ_{ik} is a systematic, observable and explainable component which is a function of the attributes of the alternatives, and ε_{ik} is a random, unobservable component which represents unmeasured variation in an individual's decision-making process or preferences. The probability that an individual *i* chooses the alternative *j* with a profile of attributes *k* in the conditional logit model can be solved as:

$P_{ik} = \exp(\mu_{ik})/\sum_{j=1}^{j} \exp(\mu_{ij})$

Attribute levels were coded from the level expected to be least preferred (used as the reference category) to the most preferred, therefore positive coefficients increasing in magnitude for each level were expected. The statistical significance of these coefficients relative to the reference category was also tested. Marginal rates of substitution, or the trade-offs between attributes and levels, are calculated by dividing one coefficient by another, known as the payment vehicle. In this study the reduction in risk of RA was set as the payment vehicle, and for the purposes of calculating MRS, this attribute was modelled as a continuous variable to yield a single coefficient. Dividing the coefficients of every other attribute and level by the coefficient for reduction in risk generates a MRS which can be interpreted as the magnitude of potential reduction in the risk (i.e. benefit) that people would be willing to sacrifice in order to achieve a more preferable level of another attribute (e.g. oral pill instead of intravenous infusion).

The potential uptake of a preventive treatment was predicted from the choice between the preferred treatment and 'no treatment for now' in each choice set, using a logit model with random effects (to account for repeated observations within individuals). Analysis of the choices between the preferred treatment or the 'no treatment for now' using a logit model generated coefficients consistent with those from the conditional logit model of the choice between two treatments (Fig S2). Coefficients from this model for each attribute level were then used to predict uptake of each preventive treatment.(3) To calculate uptake, the indirect value of each preventive treatment is calculated as the exponent of the sum of coefficients for the attribute levels best representing that treatment. Uptake for a specific treatment is then calculated as the indirect value of that treatment divided by the sum of the indirect value of all other available treatments and no treatment (e^0). Attribute levels best representing each of the current preventive treatments under study were based on expert opinion (MHu, KS, AF) (Table 2). As only treatment currently being studied for Phase C is hydroxychloroquine, Phase C was assumed to consist of hydroxychloroquine versus no treatment, and Phase D included all treatments currently under study and no treatment.

Heterogeneity in preferences was explored using a latent-class conditional logit model(4) to identify whether any unobservable subgroups could be identified within our group of FDRs based on their

preference structures. Latent class models assume that a latent variable, for example preferences exist but cannot be directly observed, so must be estimated. This latent variable is assumed to be discrete and divides a population into several classes. Each class is defined by a series of conditional probabilities which reflect the likelihood that variables (for example, coefficients for attribute levels) take certain values. The optimal model was selected as that which contained the number of classes which minimized the Bayesian information criterion (BIC) and consistent Akaike information criterion (CAIC).

Results of latent class analysis

The latent class analysis was able to estimate models with 2 to 5 distinct classes. The optimal model, which minimized both the Bayesian information criterion (BIC) and consistent Akaike information criterion (CAIC), was the model estimated with 3 classes (Fig S3).

The highest posterior probability of class membership was calculated as a measure of how well the model is able to differentiate between the proposed latent classes of preferences. For the 3 class model, the average (over respondents) of the highest posterior probability of class membership was 0.86 (SD 0.14), which indicates that the model is has a good ability to distinguish between subgroups with different importance for attributes when making choices. The ability of the model to make in-sample predictions was evaluated by assigning individuals to the class with the highest posterior probability of class membership and then predicting unconditional probabilities of actual choice and the probability of choice conditional on being in their assigned class. The results from this analysis are shown in table S1. The average unconditional choice probability is much higher than 0.5, which would be expected by chance (based on 2 alternatives to the choice made), and the average conditional probability is even higher than the unconditional choice probability for all classes. These statistics suggest that the model is able to describe observed choice behavior well.

Table S1: Class membership

	Unconditional probability	Conditional probability
Class 1	0.662	0.839
Class 2	0.588	0.622
Class 3	0.609	0.724

Fig S2: Comparison of coefficients from effects coded conditional logit and logit models

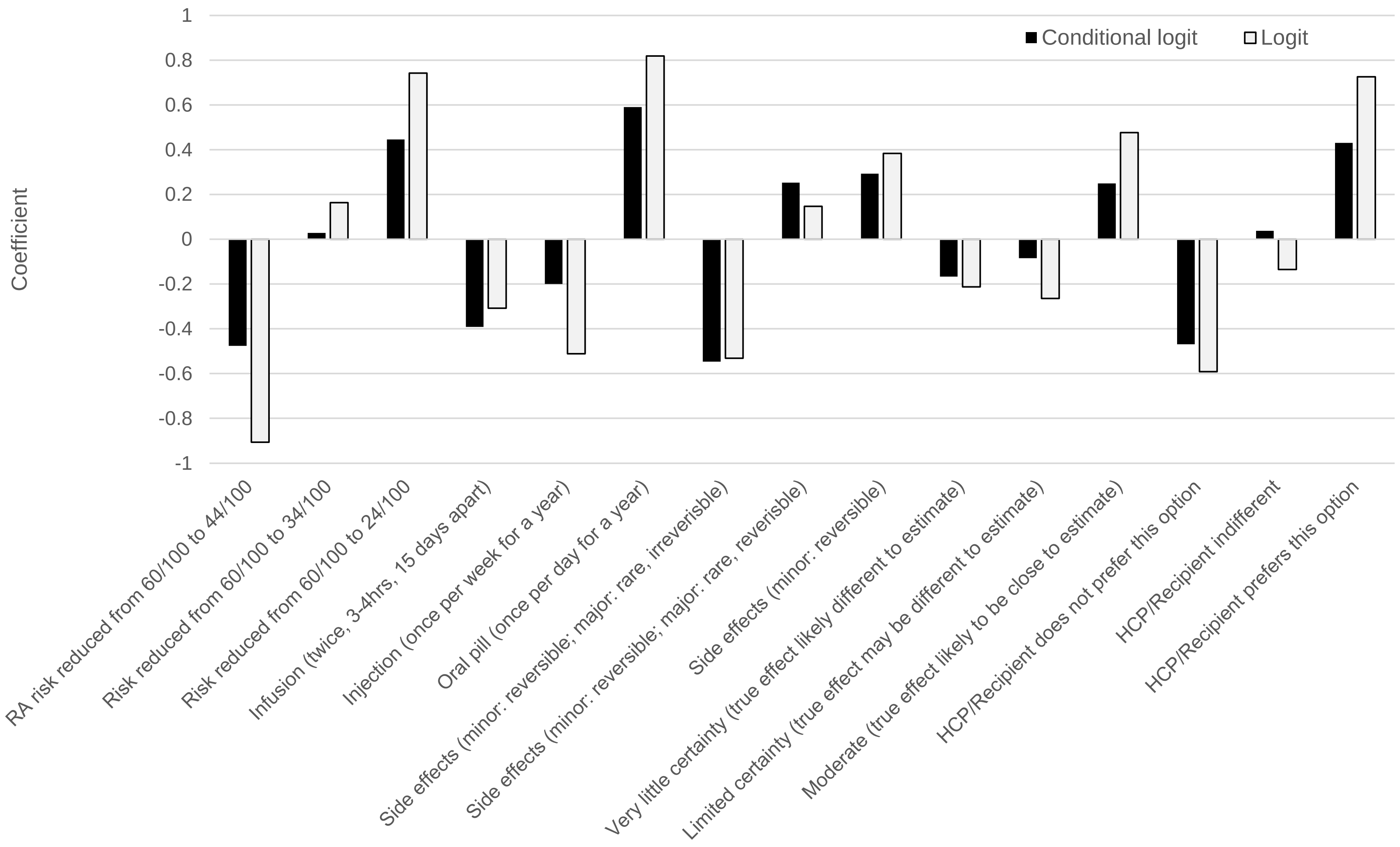

Fig S3: Bayesian information criterion (BIC) and consistent Akaike information criterion (CAIC) for models with 2 to 5 latent classes predicted.

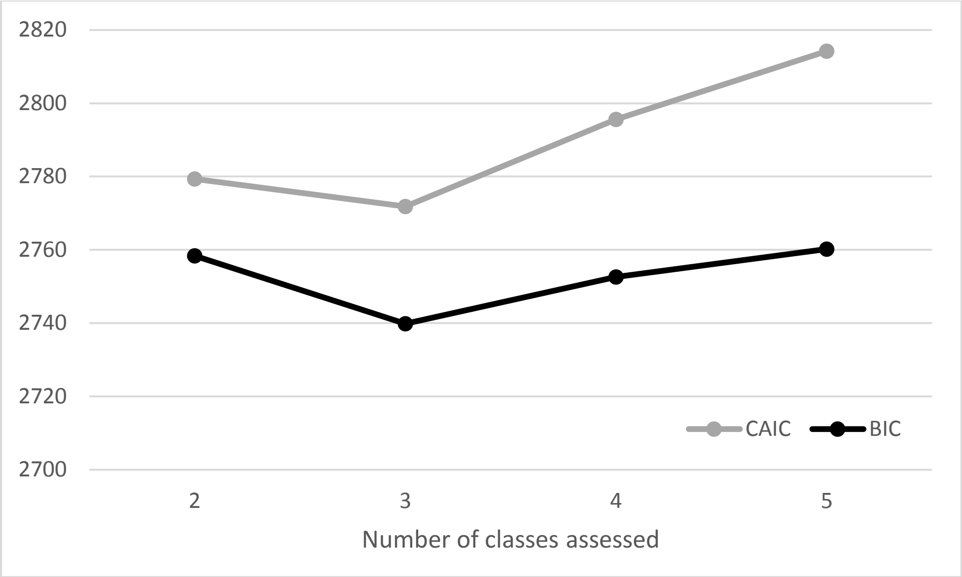

References

1. SAS. Cary, North Carolina: SAS Institute;

2. McFadden D. Conditional logit analysis of qualitative choice behavior. In: Frontiers in Econometrics. New York: Academic Press; 1974. p. 105–42.

3. Watson V, Ryan M, Watson E. Valuing Experience Factors in the Provision of Chlamydia Screening: An Application to Women Attending the Family Planning Clinic. Value in Health. 2009 Jun 1;12(4):621–3.

4. Pacifico D, Yoo HI. lclogit: A Stata Module for Estimating a Mixed Logit Model with Discrete Mixing Distribution Via the Expectation-Maximization Algorithm [Internet]. Rochester, NY: Social Science Research Network; 2012 Jul [cited 2017 Dec 21]. Report No.: ID 2205054. Available from: https://papers.ssrn.com/abstract=2205054
